# The human immunome in the post-schistosomiasis mass drug administration era

**DOI:** 10.1371/journal.pntd.0014084

**Published:** 2026-03-16

**Authors:** Emilee J. Benos, Francisca Mutapi

**Affiliations:** 1 Institute of Immunology and Infection Research, University of Edinburgh, Ashworth Laboratories, Edinburgh, United Kingdom; 2 National Institute for Health Research, Tackling Infections to Benefit Africa (TIBA) Partnership, The University of Edinburgh, Ashworth Laboratories, Edinburgh, United Kingdom; Weill Cornell Medical College, UNITED STATES OF AMERICA

## Abstract

Helminths have co-evolved with humans and developed sophisticated mechanisms to manipulate the host immune system, allowing them to persist for years. During chronic disease, helminths typically shift immune responses toward a Th2 profile—supporting antibody production and tissue repair—while suppressing Th1/Th17 responses that are crucial for combating viruses and intracellular pathogens. Additionally, they elevate regulatory T cells and anti-inflammatory cytokines such as IL-10 and TGF-β, dampening inflammation, compromising host immunity to other infections, and, in some cases, reducing vaccine efficacy. Further, both experimental and clinical studies have shown that anthelminthic treatment can reverse parasite host immunomodulation. However, there is evidence that helminth-induced immune changes may persist months after parasite clearance. With the widespread rollout of preventive chemotherapy via mass drug administration (MDA) across the continent, many African populations have now received at least one round of deworming treatment. This raises critical questions about the nature, persistence, and public health significance of anthelminthic treatment-related immunological shifts in endemic settings with repeated exposures. In Africa, which bears a disproportionate share of the global helminth burden, there is growing interest in how these factors shape immune responses. In this review, we summarise current knowledge and key research gaps regarding mechanisms that contribute to immune variation in helminth-endemic populations and the broader implications for disease control, vaccine response, and health policy in endemic settings.

## I. Introduction

The human immune system is diverse and dynamic and determines how an individual responds to natural immunogens and interventions. Many factors influence the human immune system’s response to novel infections and interventions, including individual genetics, environment, age, sex, previous infections and interventions, and co-infections. These factors exhibit complex interdependencies confounding, modulating, and amplifying one another, thereby contributing to observed inter-individual and population-level immune heterogeneity [1].

Immunoepidemiological studies have provided evidence of altered immune responses to infections and interventions in African populations, particularly in rural settings, compared to higher-income and/or urban settings. For example, there is mounting evidence of lower vaccine efficacy and impaired vaccine-specific immune responses in African settings, including for yellow fever [2], BCG [3,4], rotavirus [5], influenza [6], tetanus [7], investigational malaria [8], Ebola [9], viral-vectored tuberculosis [10], and oral polio myelitis [11,12] vaccines. Additionally, many studies showed a relatively lower SARS-Cov-2 disease burden in African countries compared to other regions [13,14].

Potential explanations for these altered immune responses on the African continent include poor nutrition, genetics, microbiome differences, younger population demographics, and underreporting [15–19]. Environmental and lifestyle factors, pre-existing immunity, and early and diverse pathogen exposure must also be considered to account for the full picture of immune heterogeneity among the African population [1].

**Fig 1:**
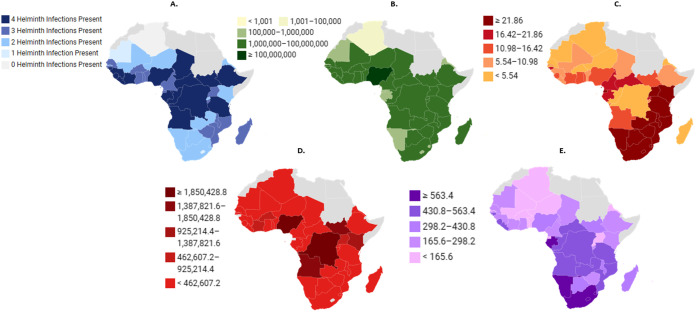
Patterns of infectious disease in sub-Saharan Africa (SSA). This figure illustrates the overlapping patterns of helminth infection and the infectious diseases responsible for the largest amount of DALYs* in SSA. This figure shows **(A)** the number of different helminth types present [24], **(B)** the number of people requiring interventions for NTDs [25], **(C)** HIV* prevalence rate per 1000 [26], **(D)** estimated number of malaria cases [27] and **(E)** TB* prevalence rate per 100,000 [28]. Data visualised using Datawrapper platform: (base layer of the maps: (A) https://www.datawrapper.de/_/wESGh/?v=2 (B) https://www.datawrapper.de/_/IsSiv/?v=2 (C) https://www.datawrapper.de/_/XHUih/ (D) https://www.datawrapper.de/_/iseqv/?v=2 (E) https://www.datawrapper.de/_/rKcZG/). **Abbreviations**: DALYs, disability-adjusted life years; HIV, human-immunodeficiency virus; TB, tuberculosis.

Co-infections are particularly common in sub-Saharan Africa (SSA) and play a major role in shaping the host response to new infections and interventions [20]. Co-infections have been shown to alter disease susceptibility, epidemiology, pathogenicity, and thus the morbidity and mortality typically associated with a single disease [21–23]. Many infectious diseases share risk factors like lack of safe water, poor sanitation, and socioeconomic disparities, which increase the likelihood of simultaneous exposure to multiple pathogens [21]. In SSA, these shared risk factors are predominant and lead to overlapping patterns of infectious disease (Fig 1) [21].

According to Woolhouse and Gowtage-Sequeria, there are 1,407 recognised species of human pathogen across the taxonomic groups of bacteria, fungi, helminths, protozoa viruses and prions [29]. Of these, helminths have received a lot of research interest as both experimental and human studies have shown that they exert profound immunoregulatory effects on the host immune system [30–35]. Soil-transmitted helminths (STH) and schistosomiasis together affect millions globally, with over 1.5 billion people impacted by STH [36] and 264.3 million requiring preventive chemotherapy (PC) for schistosomiasis, the majority of whom reside in SSA [37].

Prior infection with one pathogen can reshape immune responses to a subsequent unrelated pathogen—a phenomenon known as heterologous immunity [38]. For example, the Th2/regulatory immune profiles driven by chronic helminth infection may blunt the Th1-dominated responses required for effective vaccine or anti-viral/anti-bacterial immunity [31,32,39–45]. In tropical, helminth-endemic settings this may manifest as reduced vaccine immunogenicity [46] or altered outcomes of unrelated infections [47]. While such cross-reactive or bystander immune modulation can sometimes enhance protection, more often in these settings it appears to impair optimal immunity to heterologous challenges.

Further compounding this complicated picture of African immune heterogeneity is the success of mass drug administration (MDA) programmes. Most individuals in SSA will receive at least one round of anthelminthic treatment throughout the course of a lifetime due to widespread MDA programmes. The success of these MDA programmes raises critical questions regarding treatment-related immunological shifts, particularly in the context of reinfection events in helminth-endemic areas.

The knowledge to date on how factors characteristic of endemic helminth infection contribute to immune variation in the region are often studied in a silo, and rarely in real-world contexts that explore how they may coexist and interact within a host to create these phenotypic immunological changes. As a result, their cumulative impact on population host immunity and host responses to unrelated infections and interventions remains a critical gap in global health research.

Thus, the aim of this review is to summarise current knowledge, research gaps and potential future directions to further understand how features of endemic helminth infection—that is, chronic helminth immunoregulation and at least one round of anthelminthic treatment as a result of widespread MDA—contribute to observed immune heterogeneity among SSA populations. We also discuss the implications of those changes on how the population responds to new infections and interventions, with the ultimate goal of contextualising evidence to real-world helminth-endemic settings like SSA.

## II. The host immune profile during helminth infection

Although helminth species have distinct differences in life cycles, tissue tropism, and thus clinical outcomes, they share the ability to establish chronic infections by modulating the host immune system to evade clearance [30–35]. Helminths’ ability to suppress pro-inflammatory immune responses in these chronic conditions can lead to long-term immunoregulatory effects [31,48]. Individuals in helminth-endemic areas, like SSA, are subject to this sustained helminth-mediated immunomodulation, yet its impact on human host immune responses is not yet fully understood.

**Fig 2:**
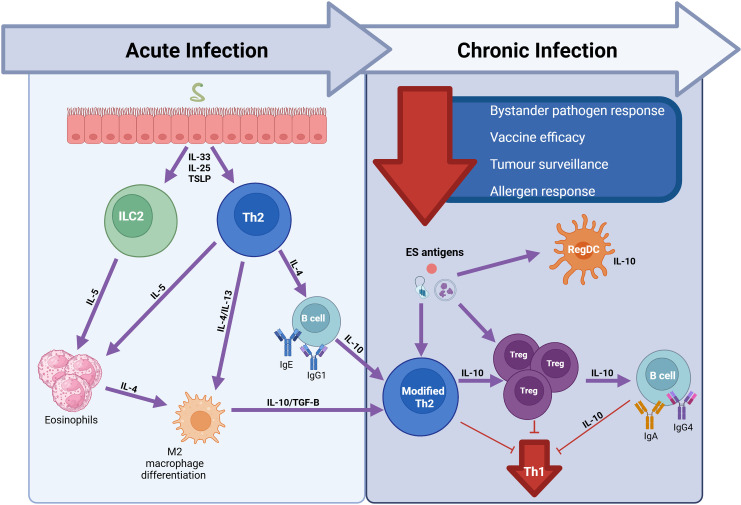
Immune profile during acute and chronic phases of helminth infection. Murine models show that in the beginning stages of infection, alarmins, including interleukin-33 (IL-33), interleukin-25 (IL-25)-producing tuft cells, and thymic stromal lymphopoietin (TSLP) are secreted by epithelial cells. These alarmins are responsible for the activation and differentiation of polyfunctional CD4 T helper 2 cells (Th2) and type 2 innate lymphoid cells (ILC2), which in turn lead to secretion of cytokines including IL-4, IL-5, and IL-13 that are responsible for eosinophilia and M2 macrophage differentiation, along with high antigen-specific IgE and IgG1 levels. This acute phase of infection is typically characterised by a pro-inflammatory allergy-like immune response. However, the continuous exposure to helminths and their excretory/secretory (ES) products in an endemic setting leads to a modified Th2 response and expansion of Treg and regulatory dendritic cells (regDCs), as well as B cell class switching to IgG4. This regulatory environment is associated with downmodulation of the Th1 response and is characteristic of chronic, asymptomatic helminth infection and leads to suppression of immunity to bystander pathogens, vaccines, allergens, and immune surveillance [39]. Created in BioRender. Benos, E. (2026) https://BioRender.com/411o63q.

Host immune profiles during helminth infection vary according to disease stage [39,48–53] (Fig 2). Helminths have complex life cycles during which several distinct developmental and migratory processes occur. Helminth infection typically occurs through host ingestion of eggs or larvae or via skin penetration and the parasite develops as it migrates through the host’s organs. The parasites mature into adult worms within a specific anatomical habitat that reflects its tissue tropism. Infection intensity or worm burden is another factor that determines host immune profiles. Due to the complex developmental and migratory stages of helminths, a host might be exposed to multiple life cycle stages simultaneously—especially in endemic settings where reinfection is common [53–56]. Other factors that determine host immune profiles include helminth type [40,57], host micro/macro environment [48,58], and excretory–secretory (ES) products of the parasite [31,59].

Helminths typically induce a mixed profile of Th1 and Th2 cytokines during the acute stage of infection. The acute phase is also characterised by high antigen-specific IgE and IgG4 levels, peripheral and tissue eosinophilia, and expanded alternatively activated macrophage populations [60,61]. This type 2 response occurs at the time of patency for adult females in peripheral blood [62,63], resulting in substantial Th1 response modulation [39].

During the chronic stage of infection, helminths tend to induce Th2/regulatory-skewed immune responses to ensure their survival in the host and minimise tissue damage. This chronic stage is characterised by the induction of anti-inflammatory Th2 cytokines including IL-4, IL-5, IL-9, and IL-13, regulatory T cells (Treg) involving cytokines like IL-10 and TGF-β, or a mix of both [33,60,61,64]. It is also distinguished by increased antigen-specific IgG4:IgE ratios and decreased antigen-specific lymphocyte proliferation [65–68] (Fig 2).

A recent study showed an early pro-inflammatory Th1-based response 4 weeks after schistosome infection, most readily apparent in symptomatic individuals with acute schistosomiasis. By week 8, an expansion of anti-inflammatory Th2 and regulatory cells were observed in both symptomatic and asymptomatic individuals, characteristic of chronic schistosomiasis [31,39,69].

## III. Helminth infection alters host responses to bystander immunogens

The chronic, nonspecific helminth immunomodulation experienced by the majority of people living in helminth-endemic areas like SSA may result in broader spill-over implications that affect systemic immune responses to bystander immunogens. There is a growing body of evidence linking helminth co-infection to altered health outcomes in SSA (Table 1). Both positive and negative outcomes of helminth co-infection have been found across studies and diseases, illustrating the complex nature of the interactions [47]. For example, evidence shows that the severity of malaria is compounded by concurrent schistosomiasis infection, and that *S. haematobium* reduces the level of protective IgG responses to potential malaria vaccine candidates, potentially undermining vaccine efficacy [70]. Helminth co-infection has also been linked to reduced severity of viral respiratory infections including COVID-19 [71–74], influenza [75,76], and respiratory syncytial virus [77,78].

**Table 1:** Summary of publications on the impacts of helminth co-infections in sub-Saharan Africa. Literature review (PubMed) using electronic search terms (a) [(co-infection* OR Coinfection*) AND (Co-morbid* OR Comorbid*) AND (Africa*) AND (Health Impact*) AND (Helminth*)] (b) [(co-infection* OR Coinfection*) AND (Co-morbid* OR Comorbid*) AND (Africa* OR Sub-Saharan Africa* OR subsaharan Africa*) AND (Health Impact*) AND (Helminth*)] (c) [(co-infection* OR Coinfection) AND (Co-morbid* OR Comorbid*) AND (Helminth*)].

Co-infections	Health impacts	Study region	Reference
Soil-transmitted helminths—Malaria	Minor impact on malaria severity	Southern Ethiopia	Degarege and colleagues [79]
Schistosomiasis—Malaria	Higher malaria parasite density in peripheral blood	Zimbabwe	Sangweme and colleagues [80]
Soil-transmitted helminths—podoconiosis	Increased anaemia and blood loss	Southern Ethiopia	Taye (2013) [81]
Helminths, HIV, or malaria coinfection in TB patient household contacts	No increased TB risk; Th1 cytokine responses reduced in individuals with prior BCG vaccination	Uganda	Biraro (2014) [82]
Helminths—Malaria	Undernutrition; severity comparable to single infections	Southern Ethiopia	Degarege (2014) [83]
Hookworm—HIV	Lower CD4+ T cell counts in antiretroviral therapy	Uganda	Morawski (2017) [84]
Urogenital Schistosomiasis—Malaria	Moderate anaemia	Northern Ghana	Dassah (2022) [85]
*Ascaris lumbricoides*—COVID-19	Negative association between ascaris lumbricoides seropositivity and COVID-19 severity	Benin	Adjobimey and colleagues [74]
*S. mansoni*, soil-transmitted helminths, malaria and hepatitis B virus	Significant association between infection status and anaemia and undernutrition	Western Ethiopia	Assefa and colleagues [86]
Hymenolepis nana, Schistosoma mansoni, Trichuris trichiura—COVID-19	Parasite co-infection associated with reduced risk of severe COVID-19	Ethiopia	Wolday and colleagues [72]
Intestinal helminths—Malaria	Associated with increased risk of malaria-associated anaemia	Southern Ethiopia	Tuasha and colleagues [87]
Helminths—tuberculosis	Clinical characteristics and co-infection patterns differ in TB patients in urban vs. rural settings	Tanzania	Sikalengo and colleagues [88]
Filarial infection—Malaria	Lower frequency of malaria-specific Th1 and Th17 T cell responses	Mali	Metenou and colleagues [41]

Numerous recent studies have also focussed on the influence of helminth infections on metabolic diseases. Many studies have found an inverse relationship between helminth infection, particularly schistosomiasis, and metabolic disease markers [66]. However, results remain heterogenous and show different effect directions depending on helminth species, infection intensity, or HIV status [67].

Helminth infection has also been linked to reduced prevalence and severity of inflammation-associated disorders like arthritis [89–91], inflammatory bowel disease [92], autoimmunity [93,94], and atopic allergy [95,96]. The nature of the relationship between helminth infection and atopy, in particular, is widely debated. The hygiene hypothesis posits that some infections, particularly in early childhood during peak immune system development, protect against inflammatory disorders [96–98]. In the context of helminth infections, the anti-inflammatory/regulatory processes triggered during chronic infection can modulate immune-mediated effector responses, thereby protecting against inflammation [96]. However, further evidence from African human studies on helminths’ role as major drivers of the hygiene hypothesis are inconsistent.

Helminths clearly modulate allergic sensitisation and immune profiles, but their role in explaining protection from asthma and allergy in Africa appears context-specific and rather modest. For example, evidence across multiple African settings links chronic high-burden helminth infections to lower skin-prick test reactivity and varying levels of allergen-specific IgE (asIgE) [96,99]. However, the associations with clinical asthma or eczema are weaker and sometimes in the opposite direction, as shown in evidence of *Ascaris lumbricoides* as a risk factor for asthma [100,101].

The heterogeneity in results illustrates the sensitive balance present in helminth-induced host responses [80].

Both helminth infection and allergy share similar immunological characteristics: elevated IgE levels, increased Th2 cell numbers, higher mast cell counts, and eosinophilia [102–104]. These similarities may be responsible for the observed effect of helminths on atopic responses, in that helminths may facilitate allergic sensitisation via IgE cross-reactivity [105,106]. Indeed, helminth-specific IgE has been shown to cross-react with allergens [106–109]. One study also demonstrated that while anti-mite IgE responses decreased post-treatment, levels of total IgE did not significantly change despite increased worm-specific IgE, suggesting that the asIgE may have been replaced by worm-specific IgE [110]. This IgE cross-reactivity may be responsible for the increased asIgE levels in the absence of clinical allergy manifestations observed in some African populations.

Evidence shows that the influence of helminth infection on the clinical manifestation of atopy is also dependent largely on infection intensity and parasite transmission dynamics. Indeed, one study in Zimbabwe found a negative association between current *S. haematobium* infection intensity and house dust mite sensitisation, but only in an area of high transmission. There were no associations found in areas of low-transmission or in low levels of infection, even in the high transmission area [111], although the role of low infection levels in facilitating allergic sensitisation has been shown for *Ascaris* [106]. Along with infection levels and transmission dynamics, helminth species also impacts the effect direction between studies, and can explain in part the observed discrepancies between allergen sensitisation and clinical disease in the context of helminth infection.

There have been a number of recent studies aimed at elucidating mechanisms of helminth immunomodulation. For example, one recent study in a murine model provides evidence of cytokine shifts as one such mechanism. This study showed that helminth-induced IL-10 signalling correlates with decreased effector T cell responses, promoting Th2 differentiation by regulating Th1 cells in infected tissue [112]. Another study in a murine model showed that shifting antigen-presenting cell populations correlate with impaired tolerance to dietary antigens, elucidating another mechanism of helminth-induced bystander regulation [113]. Another important mechanism of helminth immunomodulation is host microbiome alterations, which have been shown to have indirect effects on host immunity to bystander antigens [114]. Helminths’ ability to target macrophage epigenetics has also been shown to play an important role in inducing long-term impacts on type 2 immune responses [115]. Recent studies have also highlighted the role of helminth ES products and extracellular vesicles in modulating the host immune system and thus responses to bystander antigens. These studies have identified a growing number of immunomodulatory ES molecules, including lipids, carbohydrates, and proteins, that aide in inducing a modified Th2 response and a regulatory phenotype following persistent exposure over the course of infection [116–123].

There is still a large paucity of data on the mechanisms by which helminths regulate the human host immune phenotype and thus alter bystander immunity. This is partly because most mechanistic studies on helminths’ effect on the immune system are conducted in murine models. Immune responses elicited at localised tissue sites differ from what is measured in peripheral blood [124]. However, as logistical and ethical constraints make it difficult to access tissue cultures in humans, most immunological analyses are done on PBMCs. While many studies analysing localised tissue immune cell phenotype and function have been done in murine models, rarely have they analysed PBMCs. Therefore, there remains a gap in understanding of human and mouse immune responses to helminth infection [48]. For example, evidence shows that the proportion of ILC2s is decreased in infected human PBMCs compared to increased CD4+ Th2 cells [125,126] while ILC2s in lymphoid organs and tissues are increased in infected mice [127–131]. It may be that during the course of infection ILC2s migrate from the peripheral blood to localised tissues. Therefore, it is important to note the chronicity of human helminth infection in endemic areas. Murine models reflect acute helminth infection, so these results may be explained by the initial innate ILC2 response being replaced by the adaptive Th2 response [31,48].

This issue of chronicity (or, rather, the lack of it being addressed) reflects a larger contextual disparity between human and mice studies. While murine models provide valuable insight on the mechanistic effect of helminths on host immunity, most of the work has been done in sterilised, controlled environments that fail to reflect real-world conditions, which include co-infections and altered host immune environments. It is necessary to understand these variety of factors at play to bridge the gap between human and mice studies and gain a full understanding of how sustained chronic helminth exposure affects the human immune system.

## IV. MDA programmes for helminth infections

Further complicating the immunological picture of SSA is the widespread prevalence of MDA programmes administering anthelminthic treatment to all at-risk individuals regardless of infection status [132]. There are currently four different helminth neglected tropical diseases (NTDs) targeted by PC via MDA in the region: lymphatic filariasis, onchocerciasis, soil-transmitted helminthiasis (STH), and schistosomiasis [133,134]. MDA programmes for lymphatic filariasis and onchocerciasis are aimed at the population as a whole, while MDA programmes for schistosomiasis and STHs primarily target school-aged children [134].

**Fig 3:**
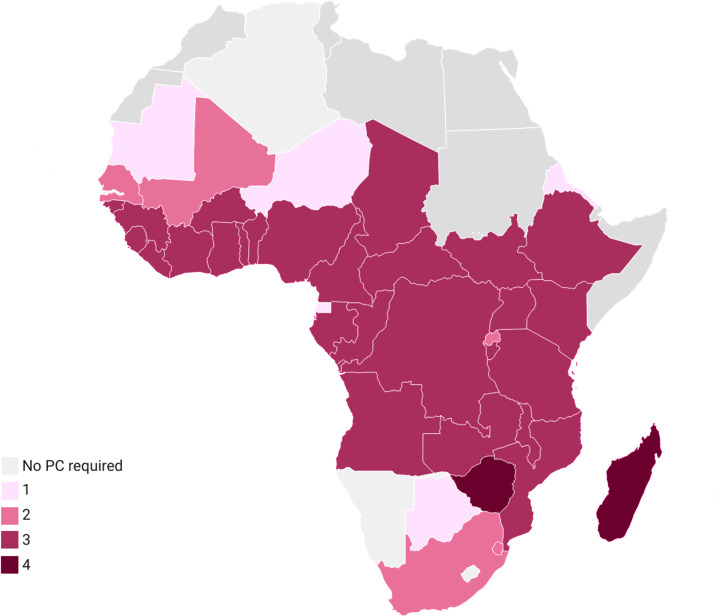
Number of anthelminthics administered via MDA, 2022–2025. This figure illustrates the number of anthelminthics administered via MDA programmes in SSA between the years 2022 and 2025. Widespread prevalence of successful MDA programmes means that most individuals in this region will receive at least 1 round of deworming treatment in their lifetime. Co-endemicity of helminth infections means that individuals in some countries may receive up to 4 different anthelminthic drugs. Data visualised using Datawrapper platform: (base layer of map: https://www.datawrapper.de/_/DYqWQ/?v=2).

The widespread proliferation and success of these MDA programmes has resulted in more individuals being treated effectively, approaching a point where the majority of people regularly exposed to helminths will have been treated with an anthelminthic at least once. Further, the MDA regimen for each country depends on the co-endemicity of other helminth infections, meaning that individuals in many countries may receive up to four different anthelminthic drugs (Fig 3). As a result, helminths will likely be encountering hosts that are not immunologically naïve, potentially affecting both helminth disease progression and host responses to unrelated immunogens [132]. Therefore, it is important to understand how anthelminthics impact the host immune system when attempting to contextualise the broader immunological implications of endemic helminth infection.

## V. The effect of anthelminthics on the human immune response

**Fig 4:**
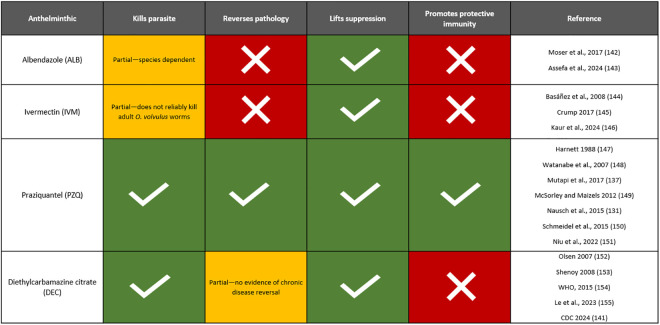
Summary of functions of MDA-administered anthelminthics. This figure illustrates the various functions and mechanisms of the four anthelminthics administered via MDA programmes in SSA. PZQ for schistosomiasis remains the most immunogenic anthelminthic.

There are several different anthelminthics available to treat parasitic infections, each with varying mechanisms of action. However, they all typically work via worm expulsion or killing of parasites stages in the host and can alter the host environment. Albendazole (ALB) or mebendazole (MEB) is used in MDA programmes for lymphatic filariasis and STH, ivermectin (IVM) for onchocerciasis, and praziquantel (PZQ) for schistosomiasis. Diethylcarbamazine citrate (DEC) is also used in MDA programmes for lymphatic filariasis, but only in areas without endemic onchocerciasis due to contraindications [134–136]. The function and effects of anthelminthics can be broken into four major pillars: its effect on the parasite themselves, its effect on disease pathology, its effect on the host immune system via lifting parasite immunomodulation, and its effect on the development of protective immunity (Fig 4).

PZQ acts immunologically synergistic with the host by reducing worm burden and promoting protective immunity [137]. This is supported by evidence from longitudinal studies that show repeated PZQ treatment boosted parasite-specific antibody responses and reduced reinfection rates [138]. Deworming for filarial infections, on the other hand, is primarily chemotherapeutic and transmission-blocking. While removal of microfilariae restores host immune competence, evidence that it boosts protective immunity in the same manner as PZQ is limited and inconsistent. Although microfilariae are cleared, evidence shows that adult worms often persist following PC and reinfection is common [139–143]. Because PZQ is highly immunogenic, we present it as a case study to further discuss how anthelminthic-induced systemic immunological changes may affect host responses to unrelated antigens.

PZQ is a broad-spectrum anthelminthic drug that has been used since the 1980s to successfully treat human helminth infections with cestodes and trematodes. PZQ has been used in schistosomiasis control programmes globally due to its low cost, ease of administration, and effectiveness [144–149]. PZQ is a pyrazino-isoquinolein derivative from the thioxantonic group [145] and acts mainly on adult worms, reducing parasite load. Its two-pronged approach accelerates host development of protective immunity by (a) removing the parasite’s immunosuppressive effects [123,137,144,150] and (b) enhancing exposure to parasite-specific antigens that induce the host immune response [144,151–154]. New parasiticidal mechanisms of PZQ continue to be elucidated, and its mechanisms of action against schistosomes have been reviewed elsewhere in the literature [144].

PZQ exhibits profound effects on the host immune system by reversing helminth-induced modulation via parasite removal. Evidence shows a change in host immune polarisation post-treatment, with an increase in Th1/Th17 responses [151], and decrease in CD4+, CD25+, and FOXP3+ Treg cells [155,156] (Table 2). Another study showed a decreased percentage of natural killer T cells in baboons after 3 doses of PZQ, and a return to baseline of CD8+ T cells after significant expansion of CD8+ over CD4+ T cell populations during chronic infection [157]. The decrease of CD8+ T cells indicates fewer parasites and thus less inflammation, as CD8+ T cells respond to egg antigens [144,158]. PZQ has also been shown to increase differentiation of Tr1 cells, which are an anti-inflammatory class of regulatory T cells that mostly produce IL-10. Tr1 cells are important to maintain homeostasis and prevent T cell-mediated diseases [154].

**Table 2:** Immune changes and outcomes post-anthelminthic treatment. This table illustrates findings from studies that investigated host immune changes that occur after helminth infection clearance via anthelminthic treatment. Most studies are experimental models or inferred in human populations. Therefore, systematic studies need to be conducted in order to determine if these changes are observed in humans, how long they are sustained, and how generalisable they are across comorbidities, co-infections, and age groups.

Immune effect	Direction of change post-treatment	Reference
Th1/Th17 responses	Increased	Bourke and colleagues [151] Maizels and McSorley [31] Wammes and colleagues [159] Toulza and colleagues [160]
Treg cells (CD4^+^, CD25^+^, FOX3P^+^)	Decreased	Elias and colleagues [161] Watanabe and colleagues [155] Correale and Farez [162] Toulza and colleagues [160] Maizels and McSorley [31] Labuda and colleagues [156]
Tr1 differentiation	Increased	Eyoh and colleagues [154]
Vaccine Response	Mixed	Wammes and colleagues [159] Elias and colleagues [161] Natukunda and colleagues [32] Nkurunungi and colleagues [163] Bourke and colleagues [164]
Allergy Risk	Possible increase	Wammes and colleagues [159] Borkow and Bentwich [165] van den Biggelaar and colleagues [166] Feary and colleagues [167]
Autoimmunity	Possible increase	Maizels and McSorley [31] WHO [168]
Inflammation	Transiently elevated	Wammes and colleagues [159] WHO [168]

However, evidence of whether anthelminthic-induced immune system changes are clinically relevant for bystander responses remain mixed (Table 2). One recent study in Uganda showed that treatment with PZQ improved BCG vaccine-specific cellular responses, but showed no significant improvements in humoral responses to any other vaccine [163]. Research suggests that helminth-induced innate immune changes, including altered monocyte and dendritic cell function, may persist months after infection clearance by anthelminthics. One study showed reversed monocyte dysfunction within 8 months following anthelminthic treatment for lymphatic filariasis [169,170]. Evidence from murine models also indicate that helminth-mediated adaptive immune changes, including memory Th2 responses [171,172], can also persist for several months after helminth clearance, although their functionality decreases without reinfection.

## VI. The population immune phenotype post-MDA: Wider public health implications

The same factors that influence the effect direction of helminth infection on immunological outcomes—namely, infection intensity, history, and transmission dynamics—also impact the immunological consequences of anthelminthic treatment and thus reflect the heterogeneity in responses. One study in Zimbabwe showed that PZQ induced more changes in anti-schistosome antibody levels in high transmission areas compared to low transmission areas [110]. Further, this study showed that anti-mite IgE decreased in adults post-PZQ, indicating a reduction in atopic responses post-treatment, particularly in high transmission areas. In contrast, results also showed an increase in anti-mite IgE and anti-worm IgE post-treatment in PSAC. Similar results were shown in a different study with IgG1 and IgA levels in school-aged children (SAC) compared to adults [173]. This evidence suggests that the post-treatment response of young children may be similar to the pre-treatment response of older people with a longer exposure history.

Taken together, these results highlight the influence of host worm burden, transmission dynamics, and cumulative exposure history in determining the immunological consequences of anthelminthic treatment. These factors must be considered when designing studies and assessing the immunological consequences of all kinds of anthelminthic treatments.

Although PZQ decreases infection intensity and alleviates morbidity, it does not prevent reinfection. Effective control programmes therefore rely on continuous administration of PZQ. However, MDA programmes run for a specified, pre-determined period, and may not be continued due to financial constraints and/or political will. It is not yet clear how long PZQ-acquired resistance lasts considering reduced natural exposure to infection to enhance protective immunity, or once the antigenic stimulation is removed due to MDA programme cessation [132].

Taken together, this evidence presents a complicated picture of immune function in areas of endemic helminth infection. Early and repeated exposure to chronic helminths infection trains the immune system towards a Th2/regulatory bias, which may explain the evidence of downregulated immune phenotypes among African populations. Chronic helminth infection has been shown to result in long-term immune imprinting, altering local immune cell populations and reprogramming haematopoietic stem cells and myeloid precursors towards an anti-inflammatory state [98,174–176]. Indeed, healthy African adults have been shown to exhibit chronic immune activation, characterised by increased levels of cytokines IL-4, IL-10, and TNF-a and low CD4+ T cell counts. This immune profile is similar to those of HIV-1 infected individuals and may contribute to observed immune hypo-responsiveness to vaccines and infections among the population [177].

Short-term immune system oscillations may also occur due to reinfection and treatment cycles in endemic areas. During chronic helminth infection, an individual may exhibit a Th2/regulatory-skewed immune profile, which may be reversed once treated with PZQ. However, the host immune profile may return to a regulatory state once reinfected. These short-term immune oscillations have important implications for therapeutic and intervention administration, as timing of these interventions will determine the immediate host immune landscape encountered, and potentially the effectiveness of the intervention. Further, the magnitude of change in immunological responses depends on infection intensity levels, transmission dynamics, helminth species, and exposure history. Taken together, these may account for the vast heterogeneity in study results among endemic populations.

These population-level immunome changes have implications for parasite-specific vaccine development. Evidence from hookworm vaccine trials show that individuals from hookworm-endemic areas exhibited higher IgE levels to the recombinant Na-ASP-2 protein vaccine target, resulting in adverse urticarial reactions [178]. Other research showed that in endemic schistosomiasis areas, host age, infection status, and PZQ treatment affected the cytokine response to the target vaccine antigen, glutathione-S-transferase (GST). In participants aged 10–12, when *S. haematobium* infection prevalence and intensity peak, GST-specific type 2 and regulatory cytokines were lower. Post-PZQ treatment, more participants exhibited GST-specific cytokines along with a shift towards a pro-inflammatory cytokine response [164] (Table 2).

Therefore, it is critical to consider the immunological phenotype of an endemic population in order to develop effective, context-specific vaccines. The potential of PZQ to alter host reactions to vaccinations should be considered when evaluating schistosomiasis vaccine efficacy, especially since the target population in endemic areas will have been exposed to PZQ at least once. The most appropriate timing for delivery of PZQ and individual vaccines must be sufficiently evaluated during clinical trials to avoid any potential hinderance to vaccine efficacy [30].

High antibody titres that remain post-treatment also raises considerations for serological schistosomiasis diagnostic development, reducing their effectiveness in endemic settings where treatment is common [179]. Further, repeated schistosome exposure in endemic regions may lead to misleading results in serological diagnostics. This could be due to decreasing antibody response with age as a result of repeated exposure [180] and/or the inability of serology to distinguish between active, past, or re-infection [181–183]. The presence of helminth co-infections in endemic settings also complicates serological diagnostics, as cross-reactivity may render it difficult to distinguish infections between helminths [184–187]. These illustrative studies reflect broader immunological implications, including how this altered immune landscape as a result of repeated schistosome and praziquantel treatment exposure may unpredictably affect vaccine and diagnostic development.

## VII. Conclusion

The overwhelmingly positive impact of MDA on helminth infection rates and related morbidity among individuals in endemic areas cannot be overstated. PC has resulted in the reduction of schistosomiasis prevalence in SAC by around 60% [144]. In fact, sustained MDA programmes have reduced schistosome infection to levels where interruption of transmission is awaiting verification [32]. The purpose of this review is not to argue against the conduct of MDA programmes, but rather understand its immunological consequences to ensure the widespread health benefits of MDA are sustained for future generations.

Little is currently known about the immune phenotype of helminth-endemic populations. Therefore, it is crucial to develop more real-world data to contribute to improved understanding of immunological differences amongst helminth-endemic populations and the collective impact of MDA programmes on the host immunological profile. Detailed mapping studies that take into account factors like infection intensity, exposure history, and transmission dynamics are needed to accurately characterise the immunological changes resulting from helminth MDA programmes. Results from these studies can help inform predictive analyses on how the populations may respond to new and emerging pathogens, vaccines, and diagnostics. Further, this data would also aid in tracking drug efficacy and risk of resistance, as well as pathology left unresolved by PC. Evidence from these studies will allow for the design of tailored, context-specific, and cost-effective public health policy and preparedness initiatives and interventions. However, existing funding gaps make this type of work extremely difficult.

The majority of clinical trials are conducted in non-helminth endemic Western populations with differing immune phenotypes. Therefore, collaborative, multidisciplinary, and targeted studies accounting for multiple disease systems are needed to develop more realistic, context-specific interventions for target populations. Screening for co-infections in early clinical trials and experimental studies can allow for the design of heterogenous trial populations representative of the population [30].

Further, the strategies and effects of helminth interventions should continually be monitored and assessed, noting the influence of varying infection intensities, exposure histories, and transmission dynamics shown through PZQ studies. Incorporating this holistic immunological monitoring into MDA impact assessments will provide insight into the effect of long-term of MDA on the trajectory of natural immunity in endemic areas. This will help sustain and maximise the substantial health benefits of MDA.

Insight gained on the effect of endemic helminth infection and treatment on a population’s immune profile will ultimately have implications for targeted control efforts and public health policymaking at local, regional, national, and international levels.

**Five key papers**

1. Human schistosomiasis in the post-mass drug administration era—The Lancet Infectious Diseases
2. Coinfections and comorbidities in African health systems: At the interface of infectious and noninfectious diseases
3. Effects of helminths on the human immune response and the microbiome
4. Peptide microarray IgM and IgG screening of pre-SARS-CoV-2 human serum samples from Zimbabwe for reactivity with peptides from all seven human coronaviruses: a crosssectional study
5. Immunological Considerations for Schistosoma Vaccine Development: Transitioning to Endemic Settings

**Key learning points**

Helminths have shaped the evolution of the human immune response and continue to alter responses against themselves and those directed to unrelated antigens.
Helminth infections are controlled through treatment of populations at risk of infection without individual diagnosis through mass drug administration (MDA) of antihelminth drugs.
In addition to killing adult worms, the drug praziquantel used for schistosomiasis MDA alters the host immune responses both to schistosomes and unrelated immunogens.
Individuals in helminth-endemic settings like sub-Saharan Africa are subjected to sustained helminth immunomodulation and at least one round of anthelminthic treatment as a result of widespread MDA programmes.
5. The systemic immune changes brought on by both the helminth infections and their treatment result in spill-over effects altering host response to bystander immunogens including unrelated pathogens, allergens, autoantigens, and vaccines.
